# Mapping pollution exposure and chemistry during an extreme air quality event (the 2018 Kīlauea eruption) using a low-cost sensor network

**DOI:** 10.1073/pnas.2025540118

**Published:** 2021-06-21

**Authors:** Ben Crawford, David H. Hagan, Ilene Grossman, Elizabeth Cole, Lacey Holland, Colette L. Heald, Jesse H. Kroll

**Affiliations:** ^a^Department of Civil and Environmental Engineering, Massachusetts Institute of Technology, Cambridge, MA 02139;; ^b^The Kohala Center, Waimea, Hawai‘i Island, HI 96743;; ^c^School of Ocean and Earth Science and Technology, University of Hawai‘i at Mānoa, Honolulu, HI 96822

**Keywords:** air quality, low-cost sensors, volcanoes

## Abstract

Extreme air quality episodes represent a major threat to human health worldwide but are highly dynamic and exceedingly challenging to monitor. The 2018 Kīlauea Lower East Rift Zone eruption (May to August 2018) blanketed much of Hawai‘i Island in “vog” (volcanic smog), a mixture of primary volcanic sulfur dioxide (SO_2_) gas and secondary particulate matter (PM). This episode was captured by several monitoring platforms, including a low-cost sensor (LCS) network consisting of 30 nodes designed and deployed specifically to monitor PM and SO_2_ during the event. Downwind of the eruption, network stations measured peak hourly PM_2.5_ and SO_2_ concentrations that exceeded 75 μg m^−3^ and 1,200 parts per billion (ppb), respectively. The LCS network’s high spatial density enabled highly granular estimates of human exposure to both pollutants during the eruption, which was not possible using preexisting air quality measurements. Because of overlaps in population distribution and plume dynamics, a much larger proportion of the island’s population was exposed to elevated levels of fine PM than to SO_2_. Additionally, the spatially distributed network was able to resolve the volcanic plume’s chemical evolution downwind of the eruption. Measurements find a mean SO_2_ conversion time of ∼36 h, demonstrating the ability of distributed LCS networks to observe reaction kinetics and quantify chemical transformations of air pollutants in a real-world setting. This work also highlights the utility of LCS networks for emergency response during extreme episodes to complement existing air quality monitoring approaches.

## Significance

Poor air quality is a global public health issue, contributing to millions of premature deaths per year worldwide. Low-cost air quality sensors are a promising tool to improve monitoring capabilities. In this study, we built and deployed a low-cost sensor network for emergency response during an extreme air quality event, the 2018 Kīlauea Lower East Rift Zone eruption. This network was used to estimate fine-scale population exposures to multiple pollutants, to measure the chemical transformation of volcanic emissions, and to provide real-time observations as part of emergency management efforts.

Outdoor air pollution leads to the deaths of millions of people per year, representing the single largest environmental risk factor for premature mortality worldwide (1). Air quality (AQ) monitoring is critical to understand and ultimately minimize people’s exposure to harmful air pollutants; however, surface-based measurements remain relatively sparse in much of the world (2). Moreover, a substantial (though poorly quantified) fraction of humans’ exposure to air pollutants occurs during extreme AQ events in which pollution levels are dramatically elevated relative to mean levels. Examples include the 1948 Donora, Pennsylvania smog event (3), the London Fog of 1952 (4), the Great Smog of Delhi (5), the Beijing “Airpocalypse” in 2013 (6), and recent severe wildfires in North America and Australia (7, 8). During such events, hazardous pollutants such as particulate matter (PM) can be primary (emitted directly) or secondary (formed via atmospheric reactions) (9). The variable confluence of primary emissions, meteorological transport dynamics, and complex secondary chemical processes makes AQ monitoring during extreme episodes exceptionally challenging. There is currently no established approach or strategy to monitor pollutant distribution or human exposure during these episodes.

**Fig. 1.**
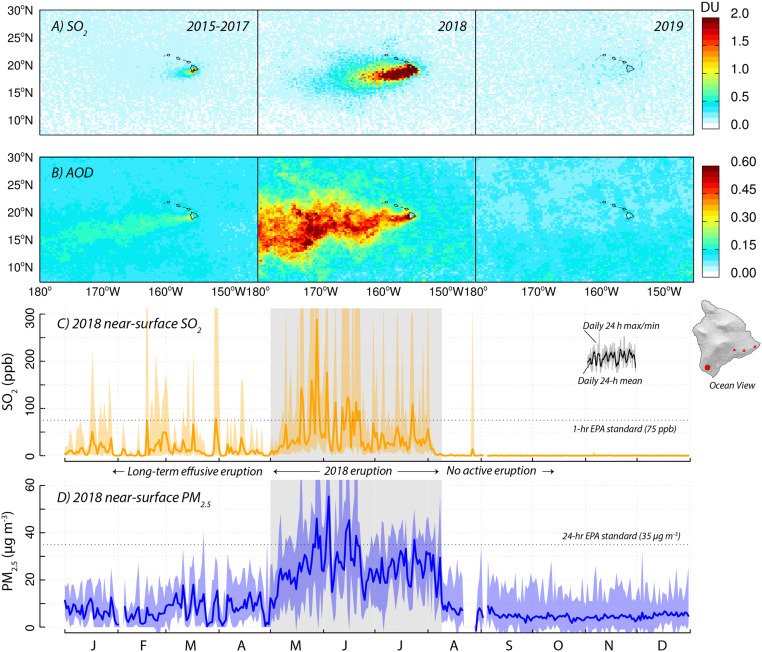
Satellite- and ground-based monitoring of air quality before, during, and after the LERZ eruption. (*A* and *B*) Satellite observations of column integrated SO_2_ and aerosol optical depth (AOD) from May to July, comparing the average of 3 y prior to the eruption (2015 to 2017), the year of the eruption (2018), and the year following the eruption (2019). SO_2_ (shown in Dobson Units [DU]) and AOD measurements are taken from the Ozone Mapping and Profiler Suite (OMPS) instrument aboard Suomi National Polar-orbiting Partnership (NPP) (50 km product, Version 2) and the Moderate Resolution Imaging Spectroradiometer (MODIS) instrument aboard the Aqua platform (10 km product, Collection 6.1), respectively. Daily satellite observations are gridded and averaged at 0.5° × 0.5° horizontal resolution. (*C* and *D*) Concentrations of SO_2_ and PM_2.5_ for all of 2018, as measured by the Hawai‘i Department of Health ground-level regulatory station at Ocean View.

Here, we track and characterize a recent extreme AQ event, the 2018 lower East Rift Zone (LERZ) eruption of Kīlauea Volcano (Island of Hawai‘i, USA, May to August 2018), using a low-cost air quality sensor network. Prior to this event, Kīlauea had been continuously erupting since 1983 (10). As the nearly constant northeasterly trade winds transported the plume downwind around the southern coast of the Island, the primary sulfur dioxide (SO_2_) emissions oxidized to form sulfuric acid, leading to elevated levels of fine PM on the island’s downwind western side (the Kona coast) (11). The SO_2_ and PM (collectively known as “vog,” for “volcanic smog”) from this effusive eruption had for decades been recognized as a local AQ nuisance and health hazard (12, 13) for the island’s ∼175,000 residents and even for residents in neighboring islands. Prior to May 2018, both satellite measurements and a regulatory network (five stations operated by Hawai‘i Department of Health) showed levels of SO_2_ and PM that were substantially elevated above those of background (marine) air (Fig. 1).

In May 2018, the eruption entered an intense phase with Kīlauea experiencing its largest rift eruption in more than 200 y (14). On May 3, eruptive fissures opened in a residential neighborhood in the LERZ, pumping out lava and emitting substantially elevated amounts of SO_2_ (>50,000 tons a day^−1^) (14) directly into a populated area. During the course of the 3-mo–long eruption, lava covered 35.5 km^2^ of land, more than 700 homes were destroyed, and thousands of residents were displaced. The elevated SO_2_ emissions led to exceedingly poor AQ not only in the immediate vicinity of the eruption but also across the wider region. The order-of-magnitude step change in SO_2_ emissions and resulting secondary PM was clearly visible from space (Fig. 1 *A* and *B*) and was also measured by the ground-based regulatory network (Fig. 1 *C* and *D*).

The measurements in Fig. 1 established the LERZ eruption as an extreme AQ event, and recent studies have used satellite and in situ measurements (15–17) to explore air quality implications and plume dynamics during the eruption. For example, analysis of regulatory network data found that 24-h average PM_2.5_ concentrations exceeded US Environmental Protection Agency (EPA) AQ thresholds eight times during the eruption in certain locations, compared to zero times during the previous 8 y (17). However, such measurements are typically designed to monitor regional-scale AQ and therefore provide limited details about the fine-scale spatiotemporal distribution of air pollutants. Satellite measurements are limited both spatially and temporally (because of overpass intervals of 1 to 3 d, pixel sizes of ∼tens of km, cloud cover, and limited vertical resolution). Ground-based regulatory measurements provide improved temporal resolution and networks are strategically placed to monitor ambient AQ in populated regions but are generally not designed to monitor fine-scale exposure from dynamic plumes during extreme events. On the Island of Hawai‘i, the average resident lived ∼17 km from the nearest regulatory AQ station (closer than the United States average of 22 km), and while this network provides continuous, high-quality measurements at key locations, this is too sparse for high-resolution estimates of residents’ pollutant exposure given the high temporal and spatial variability of the volcanic plume.

## Results and Discussion

In order to complement existing measurements and provide improved estimates of the pollutants’ spatial variability, human exposures, and rate of interconversion, we built and deployed a network of low-cost sensor (LCS) nodes to measure SO_2_ and PM throughout the region. The relative affordability and small size of LCS enables many network nodes to be deployed within a small area, thereby providing distributed measurements with a much higher spatial resolution than is possible with traditional AQ networks (18–20). LCS networks have recently been deployed in different locations worldwide (20–23); while LCSs are generally less accurate and precise than regulatory AQ instruments, recent work has demonstrated that LCS calibration via colocation with regulatory-grade monitors can enable robust measurements (24–26). Our deployment during the eruption occurred in two phases. First, an initial small-scale deployment in the LERZ, enabling civil authorities and local residents to monitor SO_2_ levels and make emergency management decisions, was carried out beginning May 14. These sensors had already been built and used in Hawai‘i (24) and were among the first SO_2_ measurements in the area. Next, a total of 30 new sensor units were built (measuring SO_2_ and PM), calibrated using the regulatory stations’ measurements, and deployed throughout the Island beginning May 23. Details of the sensor unit design and calibration are provided in the *Materials and Methods* and *SI Appendix*. After this deployment, the network had one node per ∼5,800 people and the average resident lived 4.6 km from an AQ measurement (*SI Appendix*). By comparison, the US- and globally averaged distances to a regulatory-grade AQ measurement are 22 km and 220 km, respectively (2).

**Fig. 2.**
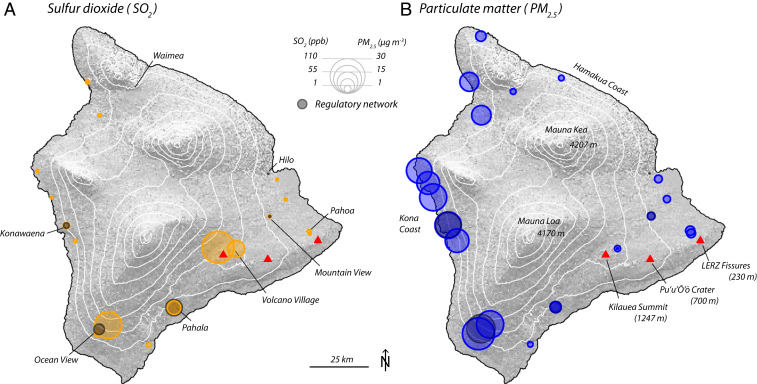
Average concentrations of SO_2_ (*A*) and PM_2.5_ (*B*), as measured by the LCS network (colored circles) and the regulatory network stations (gray circles). Data are from a 15-d period from July 15 to August 1, 2018; only the LCS nodes that were in near-continuous operation during this time are shown. For SO_2_, 17 sensors are shown, accounting for 70,414 people within 5 km. For PM_2.5_, 20 are shown, accounting for 86,856 people within 5 km. In total, there are 16 stations with both SO_2_ and PM_2.5_ measurements, accounting for 73,013 people within 5 km (Fig. 3). The full time series for all sensors are shown *SI Appendix*, Figs. S3 and S5.

Average SO_2_ and PM levels measured by a subset of the network during July 15 to August 1 are shown in Fig. 2. This subset (20 PM_2.5_ and 17 SO_2_ sensors) includes all sensors that operated nearly continuously throughout this measurement period (the full timeseries is shown in the *SI Appendix*). Both the regulatory and LCS networks measured similar overall trends in volcanic pollution downwind of the volcano; the highest SO_2_ concentrations were observed just downwind of the eruption, whereas the highest PM_2.5_ levels were further downwind, along the western (Kona) coast. LCS network stations measured peak hourly SO_2_ concentrations >1,200 parts per billion (ppb) and peak hourly PM_2.5_ concentrations >75 μg ⋅ m^−3^ (*SI Appendix*). The LCS network also revealed pollution gradients in populated areas that were imperceptible to the regulatory network, with substantial variability on finer (5 to 20 km) scales. For example, along the Kona coast, average PM_2.5_ loadings at nearby sites varied by more than a factor of two (from 9 to 25 μg ⋅ m^−3^) during the measurement period, likely because of differences in elevation, topography, and local winds. Sensors were also placed in areas that previously had been unaffected by “vog” and were without long-term monitoring stations, revealing elevated levels of pollutants there as well. For instance, in areas of the northern Kona coast, average PM_2.5_ ranged from 5 to 18 μg ⋅ m^−3^ with several short-lived episodes (<8 h) in which PM_2.5_ exceeded 30 μg ⋅ m^−3^.

**Fig. 3.**
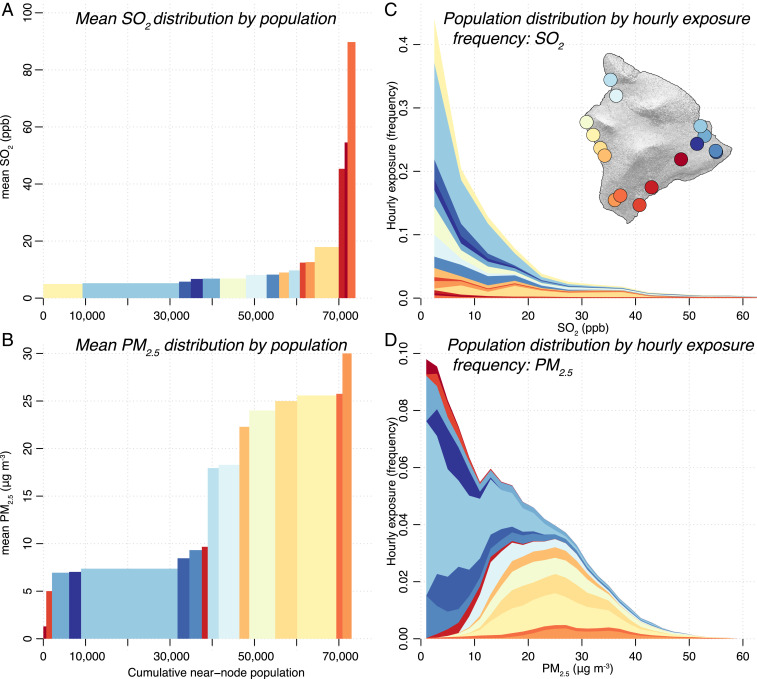
Population exposure to volcanic pollutants, measured by the LCS network over the 15-d study period. (*A* and *B*) Mean pollutant distribution as a function of cumulative near-node population (residents living within 5 km of each node: 73,013 total). Bar width is proportional to nearby population, and bar height is the average pollutant concentration measured by each node. Sensor nodes are differentiated by color, as shown on the inset map. Stations are arranged from lowest to highest average concentration. (*C* and *D*) Population distribution as a function of hourly exposure frequency to SO_2_ and PM_2.5_. Here, the distribution of hourly concentrations experienced by each sensor node is weighted by population within 5 km of the node and arranged by average concentration. Estimation of near-node population is given in the *SI Appendix*. Hourly population-weighted time series data to create (*C* and *D*) is shown in *SI Appendix*, Fig. S9. An equivalent figure using regulatory network data are shown for comparison in *SI Appendix*, Fig. S10.

The high spatial resolution of the LCS network also enables fine-grained estimates of residents’ exposure to pollutants in terms of both average exposures (Fig. 3 *A* and *B*) and hourly exposure distributions (Fig. 3 *C* and *D*). Fine-grained exposure estimates have long been viewed as a primary advantage of distributed sensor networks (e.g., ref. 18), but there exist few, if any, examples of such networks used to quantitatively estimate population-wide exposure distributions. With this sensor-based analysis (covering the >70,000 people within 5 km of a sensor node), we are able to resolve the fine structure of pollutant exposure during this extreme AQ event, analogous to previous global population-scaled annual exposure estimates based on satellite data and models (27). The choice of a 5-km buffer is somewhat arbitrary but is intended as a compromise between population coverage (*SI Appendix*) and spatial variability of point measurements as the plume chemically transforms and dynamically adjusts to underlying surface conditions. During the eruption, the highest SO_2_ levels were experienced primarily by those just downwind of the vents (Fig. 3*A*), with 5.3% of the sampled population exposed to elevated (>35 ppb) average levels of SO_2_. The LCS SO_2_ hourly exposure distribution (Fig. 3*C*) shows an extended high-concentration tail, with 2% of all hourly exposures exceeding 75 ppb (the US EPA 1-h standard, though Hawai‘i uses the pre-2010 threshold of 140 ppb). In comparison, cumulative hourly SO_2_ exposures from the regulatory network (composed of five stations covering nearly 30,000 people) are 25% of the LCS network’s cumulative total (*SI Appendix*, Fig. S9). However, the population-weighted average SO_2_ concentrations measured by the two networks are not significantly different (9.8 ppb and 9.2 ppb) because of highest SO_2_ concentrations occurring primarily in sparsely populated areas (*SI Appendix*, Fig. S8).

Exposure to secondary PM_2.5_ was more widespread; elevated average levels (>10 μg ⋅ m^−3^, World Health Organization annual standard) were experienced by nearly half (46.7%) of the sampled population (Fig. 3*B*), with 6.7% of hourly exposures exceeding 35 µg ⋅ m^−3^ (the US EPA 24 h standard). The LCS PM_2.5_ population-weighted distribution (Fig. 3*D*) also displays a clear bimodal structure, representing the overlap of spatial population and plume dynamics; a large fraction of the sampled population (mostly in the Hilo area) lives upwind of the volcano and therefore mostly experienced background levels of PM_2.5_, whereas population centers downwind of the vents experienced uniformly elevated PM levels over the entire course of the eruption. Because of the greater number of LCS stations in densely populated regions exposed to high levels of PM_2.5_, cumulative hourly PM_2.5_ exposures from the regulatory network during the study period are 28% of the LCS network (*SI Appendix*, Fig. S9). Additionally, the population-weighted average PM_2.5_ concentration from the LCS network is substantially higher than that of the regulatory network (12.9 µg ⋅ m^−3^ to 8.2 µg ⋅ m^−3^; *SI Appendix*, Fig. S8), demonstrating the importance of dense monitoring networks to complement existing measurements for accurate population-wide exposure distributions.

**Fig. 4.**
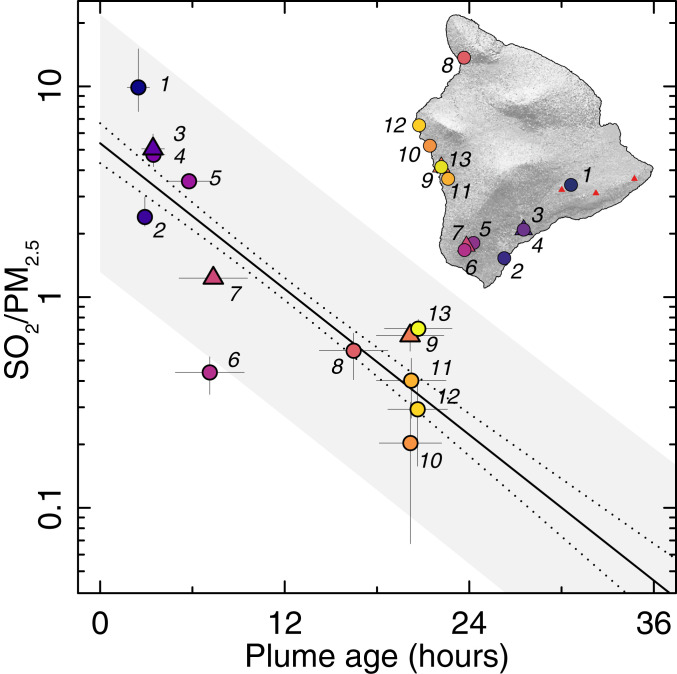
Chemical evolution of the vog plume (SO_2_/PM_2.5_ ratio), as measured by LCS nodes (circles) and regulatory stations (triangles) downwind of the volcano during the July 15 to August 1 study period. This quantifies the rate of the chemical transformation of SO_2_ (gas) to sulfuric acid (PM), yielding an estimated timescale of 7.6 × 10^−6^ ⋅ s^−1^ (lifetime of 36 h). Measurement uncertainties (vertical error bars) were determined separately for SO_2_ and PM_2.5_ sensors during instrument calibration against reference instruments (*SI Appendix*). Plume age uncertainties (horizontal error bars) are the interquartile range of model parcel travel times between the LERZ and measurement location during each hour of the study period.

The highly variable concentrations (Fig. 2) and exposures (Fig. 3) throughout the island arise not only from differences in location relative to the fissures and the plume but also from the dynamic chemical evolution of the volcanic pollutants (the oxidation of SO_2_ to form PM). Such secondary transformations of pollutants represent major challenges in AQ monitoring, which spatially dense LCS networks are well-suited to address. The high spatial resolution of our LCS network, with stations placed at different distances (i.e., plume transport times) downwind of the fissure, enables this chemical transformation to be mapped. Fig. 4 shows the evolution of the plume chemistry (described by the measured SO_2_/PM_2.5_ ratio) as a function of plume age, which is calculated using an atmospheric dispersion model (*SI Appendix*). The ratio is highest at stations closest to the eruption, with a clear decay as the plume ages.

The rate of change in plume composition (Fig. 4) implies a mean SO_2_ loss rate (*k*_*SO*2_) of 7.6 × 10^−6^ ⋅ s^−1^ (±4.8 × 10^−7^ ⋅ s^−1^) for an atmospheric SO_2_ oxidation lifetime (τ_ox_) of 36 h. While there are several uncertainties and assumptions in this calculation (see *Materials and Methods*), this value is consistent with known atmospheric SO_2_ oxidation kinetics (28) and suggests some role of not only gas phase oxidation but also conversion within cloud droplets or particles as well. Moreover, these reaction kinetics are comparable to those of other volcanoes worldwide (29), within bounds of previous measurements at Kīlauea (11, 17, 30–32), and in broad agreement with a recent estimate from the same eruption (17). Previous studies have used a range of measurement approaches—single-point, ground-based SO_2_ photometer observations (31), time-dependent downwind SO_2_ and sulfate measurements (30), satellite SO_2_ measurements (32), and SO_2_ and PM measurements from AQ stations (17)—which varied substantially in temporal and spatial coverage. The range of lifetimes of SO_2_ from Kīlauea reported in the literature, τ_ox _= ∼9 h (31) to ∼22 d (30), likely results from complex plume SO_2_ dynamics, the measurement approach used, and differences in atmospheric conditions (e.g., solar radiation, temperature, and moisture) and plume composition (e.g., aerosols, water, and oxidants). An advantage of using LCS measurements in this case is that in situ measurements over multiple days from a relatively large number of spatially distributed stations are used to aggregate highly variable plume meteo-chemical conditions. This study validates the use of distributed LCS networks for monitoring not only air pollutant concentrations but also plume chemical evolution.

In all, the LERZ eruption lasted for ∼13 wk before ending abruptly in early August 2018. After the sudden cessation of volcanic activity, AQ conditions improved appreciably across the island as SO_2_ levels fell immediately and PM_2.5_ returned to background levels after 7 to 10 d (Fig. 1 *C* and *D*). While unexpected, this extreme AQ event provided the opportunity to rapidly implement an LCS network, demonstrating the strengths of LCS for aiding in emergency response, measuring populations’ exposure to pollution, and characterizing regional atmospheric chemistry. Key features of this network were the individual nodes’ low cost, small physical footprint, stand-alone power, and real-time communications, all of which allowed for rapid, flexible deployment. Two nodes were even lost in the lava flow, highlighting the resilience of networks composed of multiple low-cost nodes.

This environment—with its small number of pollutants, point sources emitting into a clean environment, and relatively simple chemistry and meteorology—is in many ways an ideal scenario for monitoring AQ using LCS. Nonetheless, this general approach can be extended to other, more complex environments as well. The present work highlights the need for multipollutant LCS nodes that are already calibrated and readily deployable; the characterization of other extreme AQ events, such as wildfires and urban smog, requires that these nodes measure a number of additional pollutants (not just PM and SO_2_ but also O_3_, NO_2_, and CO) as well as species that can provide insight into pollutant sources and secondary chemical processes (such as NO, VOCs, and CO_2_). Furthermore, knowledge of background pollutant levels and atmospheric dynamics is necessary to isolate contributions from the event and constrain reaction kinetics. These are included in the present analysis and will be even more important in regions with higher baseline concentrations or more complex meteorology. This underscores the need for improved characterization of regional background pollutant levels (from prior regulatory, satellite, or LCS measurements) and local meteorological conditions (from wind measurements and dispersion models) in such cases. Because of the chemical complexity of wildfire and urban smog pollution, LCS measurements will also benefit from future technological improvements, such as in-line dryers to obviate the need for uncertain relative humidity corrections, and low-cost techniques for measuring PM and VOC composition to provide insight into pollutant sources, chemistry, and impacts. LCS networks thus offer the potential for characterizing pollutant exposure and chemistry under a wide range of conditions and represent an important high-resolution component of multiplatform systems to monitor and characterize extreme AQ events.

## Materials and Methods

### LCSs and Network Design.

Custom multipollutant AQ sensor (MPAQS) sensor nodes were built to measure sulfur dioxide gas (Alphasense SO_2_-B4 electrochemical sensor) and PM (Alphasense OPC-N2) concentrations, as well as auxiliary measurements of air temperature (*T*_*A*_) and relative humidity (*RH*). Additionally, six SO_2_-only nodes (24) and five PM-only nodes (Plantower PMS5003 nephelometer) were used to supplement the MPAQS network. Communications and data transmission are via 3G cellular microcontroller (Particle Electron). Sensor units are powered by rechargeable batteries and solar panels (Voltaic Systems). All sensors sample at 1 Hz and data are recorded to local storage, with 1-min averages transmitted to a custom cloud database. Total materials cost for each MPAQS node is approximately 1,000 US dollars.

The SO_2_ and PM sensors were calibrated via colocation outdoors with regulatory-grade monitors run by the Hawai‘i Department of Health (33). SO_2_ calibration algorithms are based on sensor-specific nonlinear relations to air temperature and linear sensitivity to ambient SO_2_ concentrations (*SI Appendix*). PM mass measurements are statistically corrected for ambient *RH* due to hygroscopic uptake by sulfate aerosols (*SI Appendix*). This type of field calibration approach is advantageous because instruments are exposed to dynamic and realistic ambient environmental conditions, which can be challenging to achieve under controlled laboratory settings (e.g., ref. 24).

Sensor nodes were placed primarily at schools, public health clinics, and community centers in residential areas, and each individual setting and placement was unique according to specific site characteristics and constraints (*SI Appendix*). Nodes were placed 3 to 15 m above ground level on rooftops (on tripods, electrical masts, etc.) or in open areas on facility grounds (on masts, tree trunks, and utility poles). Although sensors were deployed to more than 30 locations around the Island (*SI Appendix*), several nodes experienced intermittent communications interruptions, power issues, or sensor component failures (in particular, several SO_2_ electrochemical sensors failed, likely because of high ambient humidity). In total, there were 16 locations during the 2-wk study period with sufficient data quality and continuity for both SO_2_ and PM_2.5_ (Figs. 2 and 3; 16 locations with PM_2.5_ and SO_2_, 1 location with SO_2_ only, and 4 locations with PM_2.5_ only).

### Population Exposure Data and Analysis.

Spatial population data are 2015 Census Designated Place (CDP) level populations from the Hawai‘i State Data Center. The CDP polygons are rasterized assuming uniform density within each CDP (*SI Appendix*). The data are based on night-time residential population, and there are likely uncertainties based on nonresident populations (e.g., tourists and temporary workers), diurnal commuter patterns, and spatial settlement patterns (e.g., variations in housing density and informal encampments).

In Fig. 3, the 5-km buffer is intended as a compromise between spatial variability and representativeness of point measurements and population coverage. To avoid population double counting when stations are within 5 km, one half of the total number of residents living in the overlapping buffer zone is assigned to each station.

There are two methods used to calculate the 5-km population-weighted exposures shown in Fig. 3. First, total population exposure (*x*_*i*_) for each node (*i*) (Fig. 3 *A* and *B*) is calculated by multiplying the number of residents within 5 km of the sensor node (*p*_*i*_) by the mean observed concentrations ([*c*_*i*_]) of SO_2_ (person ppb) and PM_2.5_ (person μg m^−3^): $x_{i}=p_{i}\left[c_{i}\right]$. Second, time-integrated exposure distributions (Fig. 3 *C* and *D* and *SI Appendix*, Fig. S9) for each node (*x*_*t,i*_) are calculated as the summed product of population and smoothed (three-bin moving window) binned frequency counts (*j*) of hourly SO_2_ (person ppb hours) and PM_2.5_ concentrations (person μg ⋅ m^−3^ hours): $x_{t,i}=\overset{n}{\underset{j=1}{\sum }}p_{i}\left[c_{j}\right]$.

### SO_2_ to PM Conversion Rate.

The mean SO_2_ reaction rate is calculated based on observed SO_2_ and PM_2.5_ at 13 stations downwind of the eruption. The SO_2_ and PM_2.5_ concentrations are converted to mass concentrations of sulfur (*S* in units of μg ⋅ m^−3^) and the fraction of total gas phase *S* (*f*_*S*_) is

$$f_{S}=S_{SO2}/\left(S_{SO2}+S_{PM2.5}\right).$$ [1]

This procedure assumes all remaining PM_2.5_ is secondary sulfuric acid aerosols, originating from volcanic SO_2_ emissions. (The mass concentration of liquid water had been subtracted already as part of the PM RH-correction; *SI Appendix*.) This approach neglects background levels of nonvolcanic aerosols (∼5 µg ⋅ m^−3^ based on the network average PM_2.5_ measured from September 1 to 30, 2018 after the eruption had ceased). This fractional conversion is advantageous because reaction rates calculated using SO_2_ measurements alone can be overestimated due to nonoxidative SO_2_ losses from deposition or dilution (30).

An exponential decay function (λ) is then fit to the downwind measurement points:

$$f_{S}=\;f_{S0}e^{-k_{SO2}t},$$ [2]

where *t* is the mean plume travel time between the LERZ and measurement locations from July 15 to August 1, 2018, calculated using a particle dispersion meteorological model (*SI Appendix*). Here, *k*_*SO*2_ represents the first-order decay constant of SO_2_ and so the mean SO_2_ lifetime is equal to *k*_*SO*2_^−1^. To incorporate measurement and plume age model uncertainties into confidence and prediction intervals (95%) (Fig. 4), the decay function is fit to an array of random points uniformly sampled within the uncertainty bounds at each measurement point (*n* = 10 points at each location; total *n* = 130). Measurement uncertainties were determined for SO_2_ and PM_2.5_ sensors during instrument calibration against reference instruments (SO_2_ mean absolute error [MAE] is 7.3 ppb and PM_2.5_ MAE is 4.5 µg ⋅ m^−3^) and modeled plume age uncertainties are the interquartile range of calculated parcel travel times between the LERZ and measurement location during each hour of the study period (*SI Appendix*).

The plume SO_2_ conversion rate (*k*_*SO*2_) fit to all downwind stations (*n* = 13) is 7.6 × 10^−6^ s^-1^(τ_ox_ = 36.3 h), while the *k*_*SO*2_ fit to only the LCS network stations (*n* = 10) is 7.2 × 10^−6^ ⋅ s^−1^ (τ_ox_ = 38.3 h), a statistically insignificant difference demonstrating the ability of the low-cost network to observe reaction kinetics. Additionally, there is not a dramatic difference when *k*_*SO*2_ is fitted to measurements that have had approximate background PM_2.5_ levels subtracted (*k*_*SO*2_ = 6.0 × 10^−6^ ⋅ s^−1^; τ_ox_ = 46.4 h). However, *k*_*SO*2_ fit using plume ages estimated from an observed mean wind speed at a single location and downwind linear distance is 1.9 × 10^−5^ ⋅ s^−1^ (τ_ox_ = 15 h), substantially faster than the rate using plume ages calculated from the particle dispersion model. This demonstrates the importance of local meteorological variations, especially in areas of complex terrain, to estimate reaction kinetics.

## Supplementary Material

Supplementary File

## Data Availability

All study data are included in the article and/or *SI Appendix*.

## Change History

June 23, 2021: The author line has been updated.
