# Supplementary material for: Mapping pollution exposure and chemistry during an extreme air quality event (the 2018 Kīlauea eruption) using a low-cost sensor network
Source: Proc Natl Acad Sci U S A. 2021 Jun 21;118(27):e2025540118. doi: 10.1073/pnas.2025540118 (PMC8271697; doi:10.1073/pnas.2025540118)
Supplement: Supplementary File [file pnas.2025540118.sapp.pdf]

Supplementary Information for

Mapping pollution exposure and chemistry during an extreme air quality event (the 2018 Kīlauea eruption) using a low-cost sensor network

Ben Crawford[1,2*], David Hagan[1,3], Ilene Grossman[4], Elizabeth Cole[4], Lacey Holland[5], Colette L. Heald[1], Jesse H. Kroll[1*]

[1]Department of Civil and Environmental Engineering, Massachusetts Institute of Technology, Cambridge, MA, USA

[2] now at Department of Geography and Environmental Sciences, University of Colorado, Denver, CO, USA

[3]now at QuantAQ, Inc.

[4]The Kohala Center, Waimea, Hawai'i Island, HI, USA

[5]School of Ocean and Earth Science and Technology, University of Hawai'i at Manoa, HI, USA

***Correspondence to**: benjamin.crawford@ucdenver.edu, jhkroll@mit.edu

This PDF file includes:

Supplementary text
Figures S1 to S12
Tables S1 to S4
SI References

Publication data is available here:
https://www.notion.so/Crawford-et-al-2021-Mapping-pollution-exposure-and-chemistry-during-an-extreme-air-quality-event--082995e3c94746c4907c3c72e960b1f1

Supplementary Information Text

Materials

A schematic of the multi-pollutant air quality sensor (MPAQS) used in this study is shown in Fig. S1; the design is based on previous sensor nodes from our group (*1*). Each MPAQS node contains an electrochemical cell for measuring SO_2 (Alphasense SO2-B4, Alphasense Ltd), an optical particle counter (OPC) to measure PM (Alphasense OPC-N2), and one RH+T_A sensor (SHT3x-ARP, Sensirion). The sensors and electronics are contained in a waterproof polycarbonate enclosure (218.19 x 167.39 x 129.79 mm, YH-080604, Polycase Inc). Each node is powered by photovoltaic panels (2 x 9 W) and rechargeable battery (44 Wh) (Voltaic Systems Inc.). Sensors, electronics, and communications are integrated by a custom printed circuit board and the node is controlled by custom software on a micro-controller (Electron, Particle Inc.).

In addition to the MPAQS nodes, there were six prototype SO_2-only nodes (see *1* for full details) and five prototype PM-only nodes in the network. These SO_2-only nodes were used in the initial deployment near the LERZ eruption. These additional nodes also measure T_A and RH and use the same power and communications equipment as the MPAQs. The SO_2 sensors in all nodes throughout the network are identical while the PM-only nodes use a different sensor (PMS5003, Plantower) than the MPAQS. Sensor nodes were deployed to 33 locations across the Island (Table S1).

Methods

Sulfur dioxide measurements and calibration

The SO_2 sensors (Alphasense SO2-B4) are electrochemical devices in which a redox reaction generates a measurable electrical signal in proportion to the amount of SO_2 present in the atmosphere. In the MPAQS node design, the sensors are exposed directly to ambient atmosphere and passively ventilated through apertures in the node enclosure. For the prototype SO_2-only nodes, ambient air is actively drawn through a flow-tube and across sensors by a small fan (see *1* for full design details).

The electrochemical SO_2 sensors' electrical output signal is sensitive to ambient air temperature (T_A) in addition to SO_2. Sensors are calibrated by algorithm based on sensor-specific i) linear sensitivity to SO_2 concentration and ii) non-linear baseline response to T_A:

$$ppb = \frac{V - \beta}{\sigma} \qquad (1)$$

$$\beta = c + ab^{T_A} \qquad (2)$$

where *ppb* is SO_2 mixing ratio in parts per billion by volume, V is measured voltage (mV) from the 'working electrode' of the sensor, β is the temperature-dependent baseline, and σ is instrument sensitivity (mV ppb^{-1}). The baseline T_A exponential function parameters (*a*, *b*, *c*) for each sensor are determined using observed variations in sensor voltage with T_A over a training period of several days (Fig. S2). Factory-determined σ (~1 mV per 3 ppb) for each sensor was confirmed before deployment using a laboratory calibration chamber at constant temperature.

The calibration procedure was evaluated using co-located measurements with HDOH reference instruments during a 48-hour period at the Konawaena HDOH station (July 7-9, 2018, SO_2 values range from 0 – 50 ppb) and a 24-hour period at the Pahala HDOH station (May 27-28, 2018 SO_2 values range from 0-800 ppb). The reference instrument at each site is a regularly-calibrated UV fluorescence monitor (Teledyne 100E) and reference data are available online as 5-minute averages. Overall, average uncertainty for all electrochemical sensors is 7.3 ppb (average mean absolute error, MAE, of 5-minute values during the calibration period) with individual sensors' MAE ranging from 2.3-13.6 ppb (Table S2). Based on earlier measurements, sensor drift is negligible over the span of several months (*1*).
For quality control, data are flagged and withheld from analysis for three hours after the sensor is powered on to allow the sensor to warm-up. Additionally, a standard deviation filter (±5 times s.d.) is applied to the 1-minute time series to screen any unrealistic short-term spikes (0.6% of

1 min values). The 1-minute SO_2 dataset from July 15 – August 1 used for this analysis is shown in Fig. S3.

Particulate matter measurements and calibration

For PM, ambient air is continuously drawn to the sensor through an aperture in the node enclosure via a 7 cm length of conductive tubing. The MPAQS PM sensors are laser optical particle counters (OPCs) in which laser scattering by airborne particles provides information on particle number and size distributions. The sensor uses a 658 nm laser and provides particle counts in 16 size bins (0.38-17 μm). Mass concentrations (PM_1, $PM_{2.5}$, and PM_{10}) are calculated from binned counts using assumed particle shape (spherical), refractive index (1.5), and particle density (1.65 g mL^{-1}). A different type of low-cost PM sensor (a nephelometer) was used in the five PM-only nodes. The smallest particle size measured by each sensor is 380 nm (OPC) and 300 nm (nephelometer), according to manufacturer specifications.

The PM measurements from both sensors are sensitive to relative humidity (though nephelometer and OPC RH-responses are not identical (*2*)), due to water uptake by the highly hygroscopic sulfate particles (Fig. S4). To account for this, an RH correction based on κ-Köhler theory (*3*) was applied to enable direct comparison with reference instruments. The reference instruments use a beta-ray attenuation technique (Beta-ray Attenuation Monitor, Met-One Instruments) and ambient air is dried to 30% RH prior to analysis (hourly data, available online). Hygroscopic growth of sulfuric acid particles can occur at lower RH levels, however, there was good agreement with reference measurements in this environment during relatively dry conditions (RH<50%) and application of the RH correction below this threshold resulted in poorer agreement and so was not applied.

The correction factor (*f*) assumes an average bulk particle density (ρ) and hygroscopic growth factor (κ):

$$f = \frac{1 + \frac{\kappa}{\rho}}{-1 + \frac{1}{RH * 0.01}} \quad (3)$$

In this environment, ρ and κ values representative of sulfuric acid (ρ=1.84, κ=1.19) were used (*4*). As a sensitivity test, ammonium sulfate values (ρ=1.77, κ=0.53) were also applied and the resulting difference to mean $PM_{2.5}$ at the Konawaena reference station is minimal (MAE values differ by 0.7 μg m^{-3}, relative to reference).

Overall, there is good inter-instrument agreement between nephelometers (mean $PM_{2.5}$ values of individual sensors are within 0.5 μg m^{-3}), while variation between individual OPCs is greater (differences up to 5 μg m^{-3}). To minimize inter-instrument differences, a secondary linear calibration factor is applied to the LCS measurements based on comparison to reference instruments. The average adjustment to mean $PM_{2.5}$ from the linear calibration factor is 5.9 μg m^{-3} and measurements from the two sensor types (OPC and nephelometers) are statistically indistinguishable after the RH correction and linear calibrations have been applied. Overall, the average uncertainty of hourly $PM_{2.5}$ values for all sensors, relative to a reference instrument, is 4.5 μg m^{-3} (Table S2). This magnitude of uncertainty is comparable to that of the reference instrument itself in ambient conditions (*5*).

Additionally, the one-minute PM timeseries are subject to a standard deviation quality control filter (±5 times s.d.) to screen any unrealistic short-term spikes (0.5% of 1-minute values). The 1-minute $PM_{2.5}$ dataset from July 15 – August 1 used for this analysis is shown in Fig. S5.

Population data and analysis

Overall, approximately 61% of the Island's population (106,211 people) lived within 5 km of an AQ monitoring station in operation at some point during the eruption. Of this population, ~10,000 lived within 5 km of multiple stations and an additional 16,442 people live within 5 km of a PM monitor only, but not SO_2. Of the 87,400 remaining people, a majority (56%) lived upwind of the eruption and were largely unexposed to vog during the 2018 eruption (Table S4).

The population near each network node's location is determined in a GIS using circular buffers of varying radii from 0.5-10 km (Figure S6B, Tables S3-S4). To determine population coverage for the entire network, the individual buffer populations for each node are summed and any overlapping buffers are merged to avoid double-counting.

The average residents' distance (*d*) to an air quality monitoring station is the population-weighted straight-line minimum distance of each populated grid-cell (d_i) to the nearest AQ station:

$$d = \sum_{i=1}^{n} \frac{p_i d_i}{p_{tot}} \quad (4)$$

where p_i is the individual grid-cell population and p_{tot} is total population.

Additionally, population-weighted mean concentration [c_p] are calculated as the population-weighted mean of SO_2 (ppb) and $PM_{2.5}$ ($\mu g\ m^{-3}$) across multiple nodes (Figs. S7-S8):

$$[c_p] = \sum_{i=1}^{n} \frac{p_i [c_i]}{p_{tot}} \quad (5)$$

An hourly timeseries of accumulated population exposure (persons x hourly concentration) from both the LCS and regulatory networks are shown in Fig. S9. On average, the accumulated regulatory network total is 61% of the LCS network for SO_2 and 29% for $PM_{2.5}$. The difference in pollutant distributions across the population between networks is also illustrated in Fig. S10. This figure shows pollutant exposure distributions based on regulatory network observations (5 stations, with ~28,000 people total living within 5 km of the stations) using the same approach as in Figure 3 of the main text.

Meteorological model Plume ages were calculated using a custom application of the HYSPLIT dispersion model for the July 15 – Aug 1, 2018 study period. The plume model is initialized with modeled wind fields and thermodynamic data from the Weather Research and Forecasting Advanced Research WRF (WRF-ARW) Model at 900-m grid spacing for the Island of Hawai`i with hourly resolution out to 60 hours (Figure S11). Additional details of the general model configuration can be found in ref. *6.*

For this application, simulated air parcels (about one parcel per minute) were tracked backwards in time with the model for 60 hours or until they left the model domain. The HYSPLIT time-reversed runs were initialized hourly and output minutely to characterize the average path the air parcels had taken to arrive at each of the twelve stations (Figure S12). The air parcels that could be traced backwards to the LERZ and summit eruption coordinates (the dominant source of SO_2 during the eruption) were used to estimate the age of the volcanic plume. The plume ages are calculated by subtracting the start time from the time the particle (air parcel) arrives at the source. To simulate stochastic processes in the atmosphere, a random component is added to the advection component by the mean flow. During the study period, winds were predominantly from the northeast and the majority of parcels remained within the subtropical boundary layer (<~1800 m) by the trade wind inversion. During daytime, vog was carried onshore and upslope from localized sea-breeze circulations due to solar heating of the land surface.

Additionally, downwind of the Island in the lee of the prevailing flow, there is a large persistent clockwise eddy circulation (*7*) that transports vog first northward and then eastward towards the western coast. This flow pattern results in similar plume ages (20.6-21.1 hours) at the five stations along the western coast as the dispersed plume was carried ashore (Figure S12).

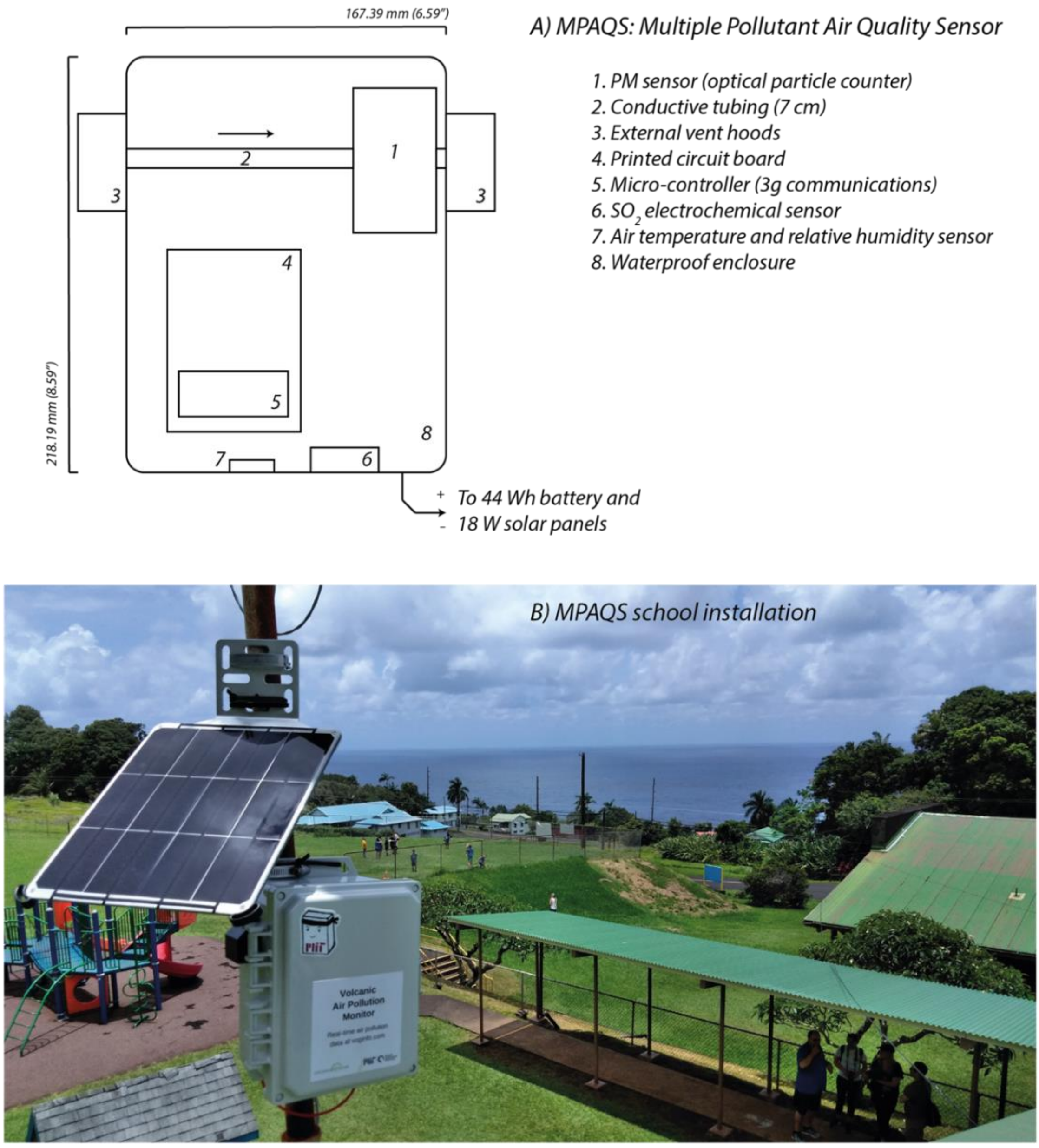

Fig. S1. Multiple pollutant air quality sensor node schematic (not to scale) and B) example field installation above a school roof in Hawai`i.

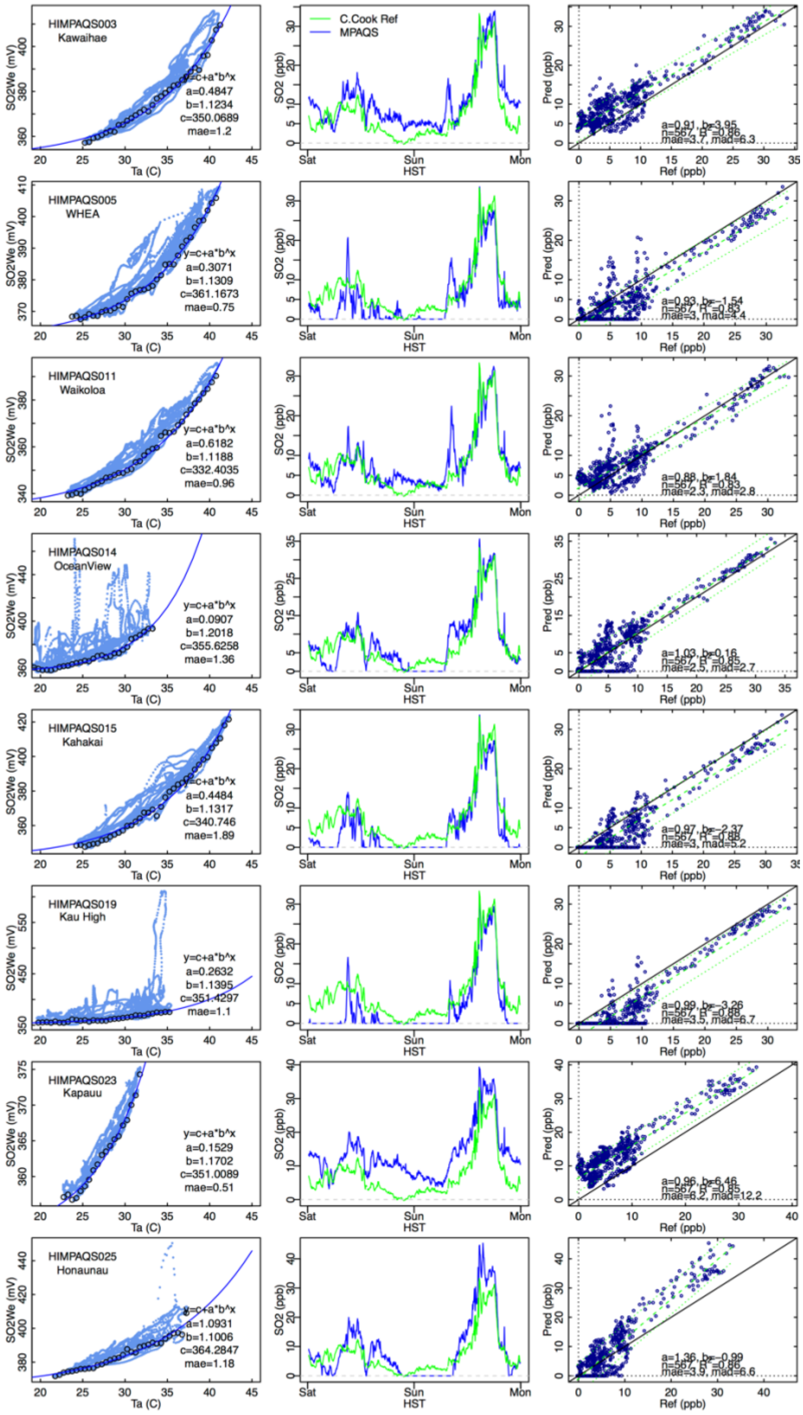

Fig. S2. Calibration procedure and statistics for eight representative SO_2 sensors. Each row is a unique SO_2 sensor. The far left column is the non-linear T_A-voltage relationship during a 7-day training period; the center column is a time-series comparison to the reference instrument co-located at the Konawaena (Captain Cook) HDOH site during the 48-hour validation period; and the far right column is a scatterplot comparison with the reference instrument during the 48-hour intercomparison period (5-minute averages).

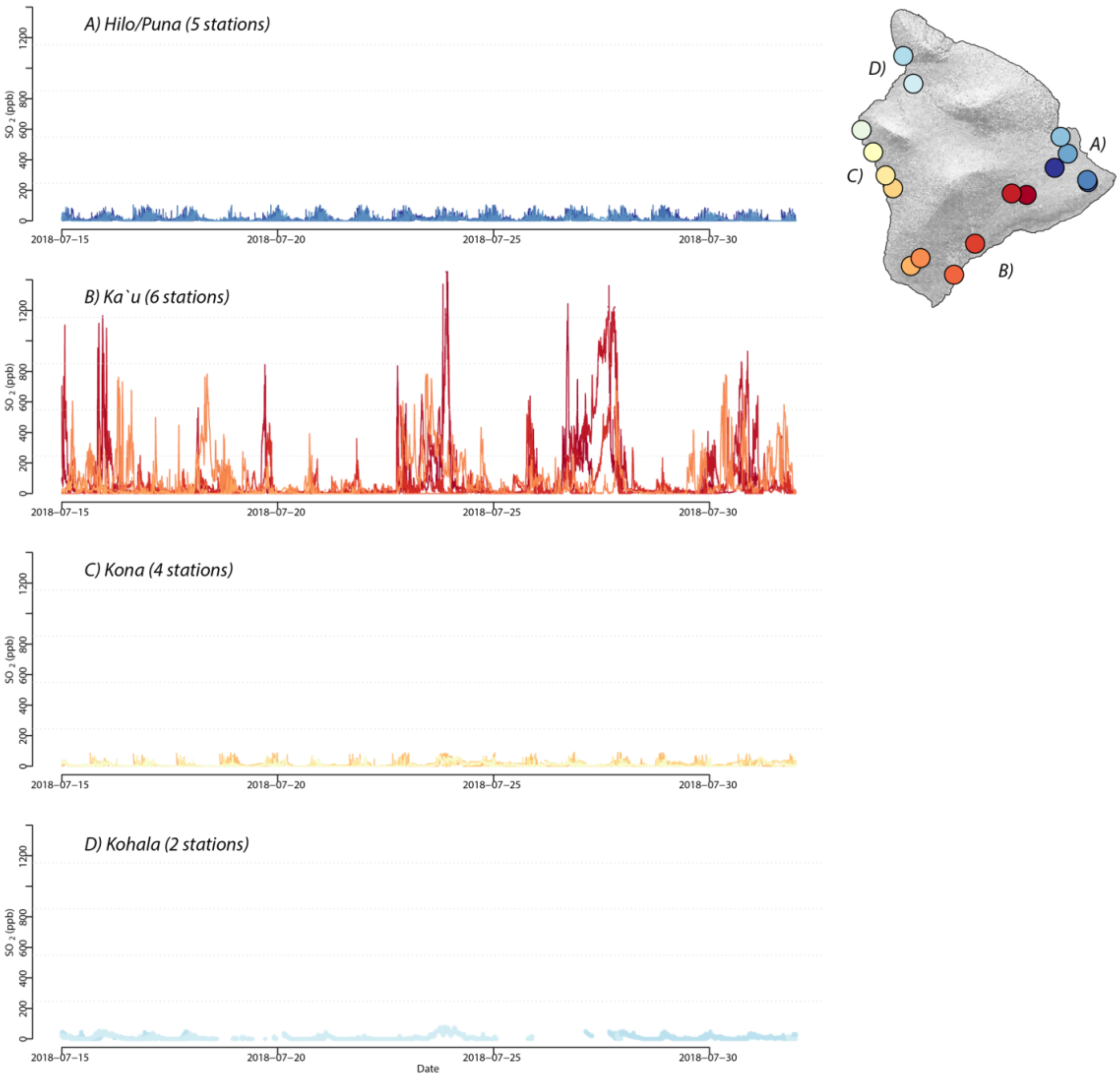

Fig. S3. Timeseries plots of 1-minute SO_2 data from the LCS network study subset (Table S2) for July 15 – August 1, 2018.

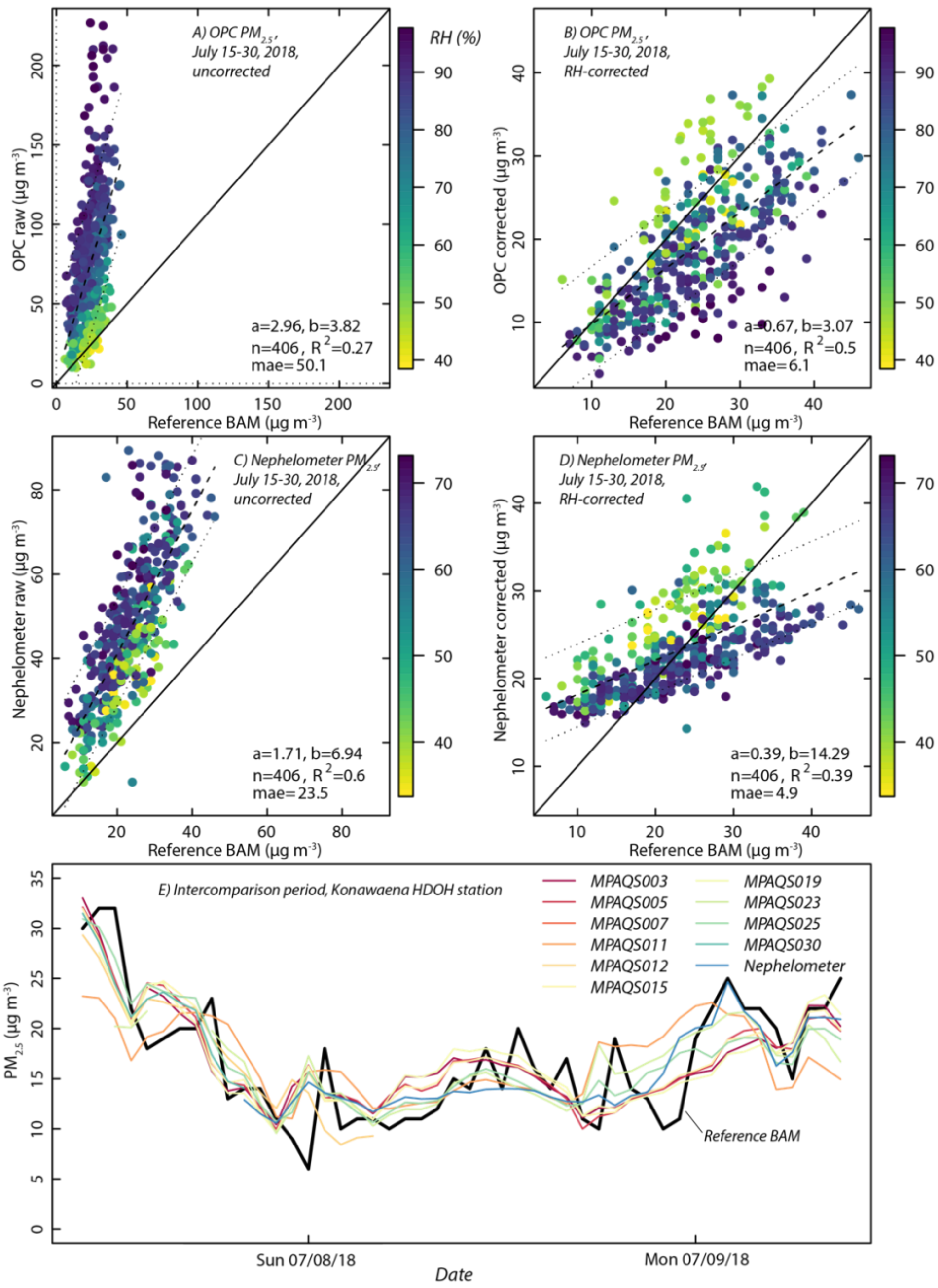

Fig. S4. Particulate matter relative humidity (RH) correction and linear calibration. Panels A-D) are hourly data from an individual LCS co-located with Konawaena reference station data (July 15-August 1, 2018). Panel A) is uncorrected (raw) LCS OPC data compared to a co-located reference instrument, panel B) is LCS OPC data that has been RH-corrected, but before the linear calibration. Panels C-D) are the same but for the nephelometer LCS model. After the RH correction, a linear calibration is applied based on comparison with the reference instrument. Panel E) shows co-located individual hourly sensor data (OPCs and nephelometer, both RH-correction and linear calibration factors applied) and the reference instrument for a 48-hour inter-comparison period at the Konawaena site. The reference sensor is a beta-ray attenuation monitor (BAM) operated by the Hawai`i Department of Health.

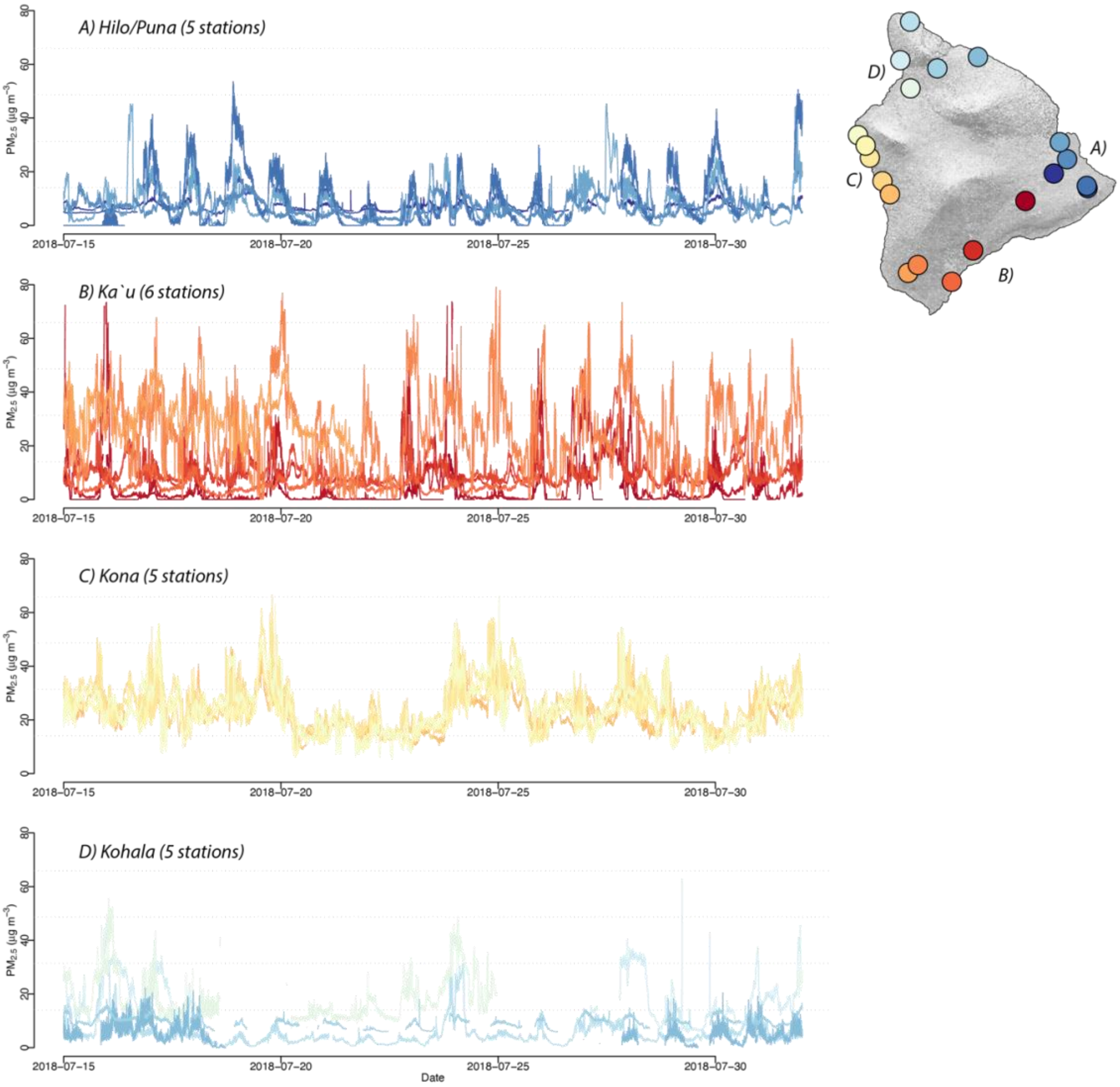

Fig. S5. Timeseries plots of 1-minute $PM_{2.5}$ data from the LCS network study subset (Table S2) for July 15 – August 1, 2018.

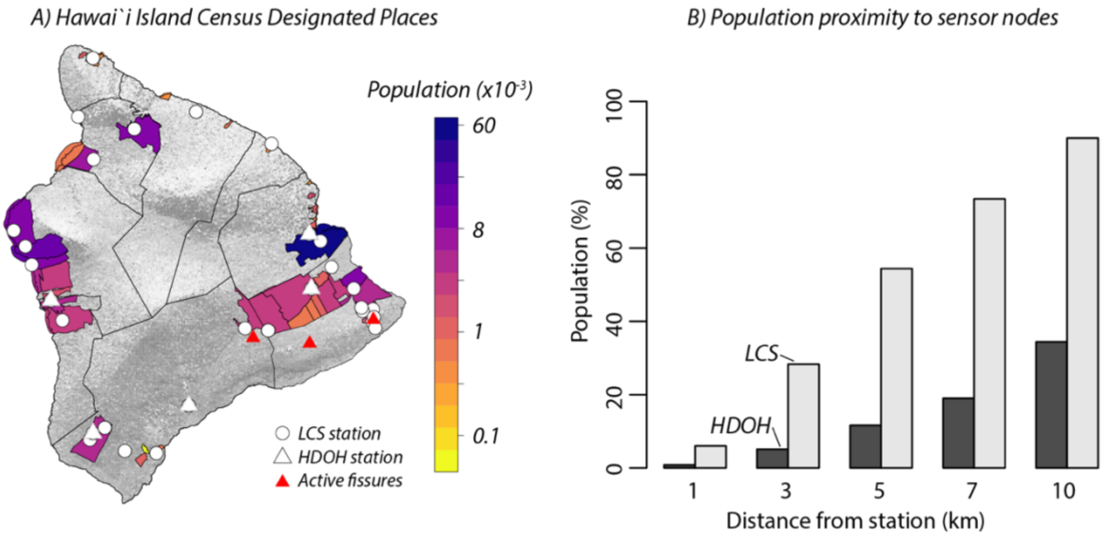

Fig. S6. A) Hawai`i census designated place population (2015) and B) resident proximity to Hawai'i Department of Health (HDOH) regulatory network and low-cost sensor (LCS) nodes. Total population of the Island is 174,256.

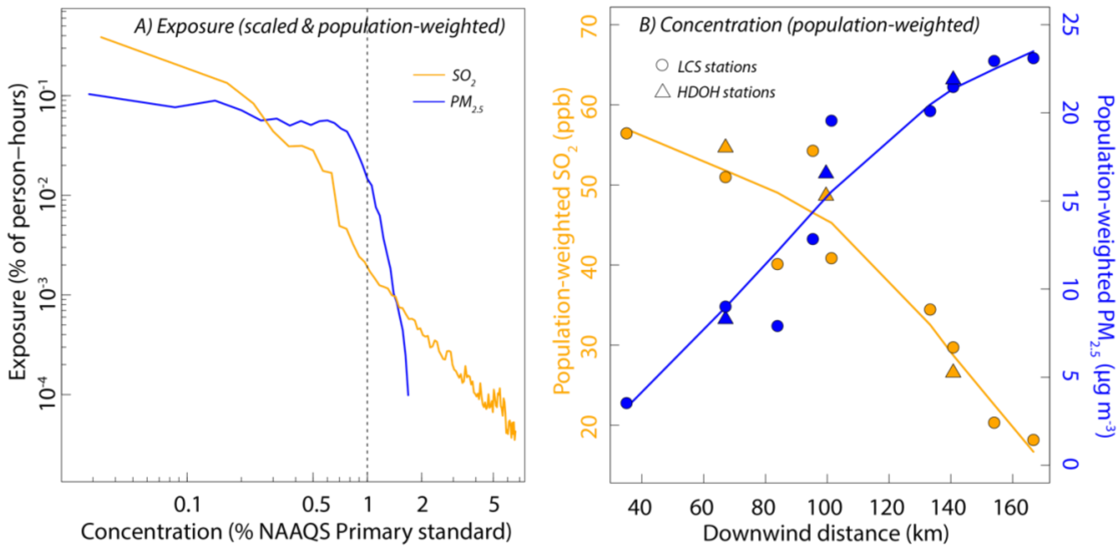

Fig. S7. A) Cumulative time-integrated exposure distribution (eq. S5) for 16 co-located SO_2 and $PM_{2.5}$ stations, scaled by US EPA NAAQS standard (24-hour $PM_{2.5}$ standard is 35 µg m^{-3}; 1-hour SO_2 standard is 75 ppb). Both axes are logarithmic. B) Cumulative population-weighted concentrations for 12 stations downwind of the eruption (eq. S7), fit with a LOWESS curve. As the plume is carried downwind it encounters more densely populated areas and chemically evolves (oxidation of SO_2 to form PM); therefore, the cumulative SO_2 population-weighted concentration decreases while cumulative $PM_{2.5}$ population-weighted concentration increases.

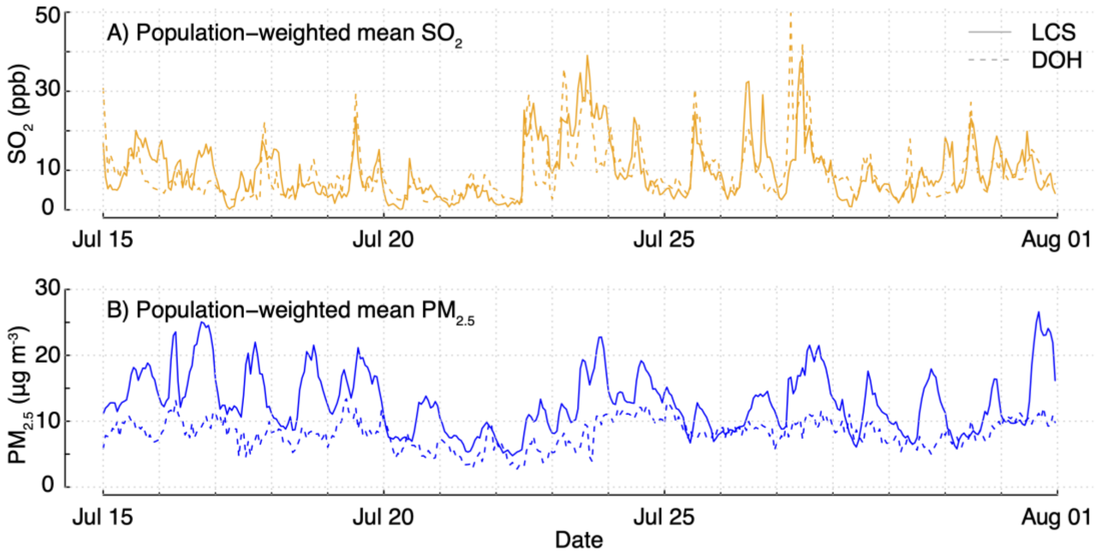

Fig. S8. A) Population-weighted mean hourly timeseries for A) 16 SO_2 sensors from the LCS network and B) five regulatory Hawaii Department of Health (DOH) sensors. Population-weighted means are calculated using Eq. S5. For SO_2, population-weighted average concentrations are similar: 9.8 ppb from the LCS network and 9.2 ppb from the DOH network. For $PM_{2.5}$, there is a significant difference in the population-weighted mean between networks: 12.9 µg m^{-3} (LCS) and 8.2 µg m^{-3} (DOH); this difference attributable to the higher density of LCS monitors in populated areas (mostly along the Kona coast) affected by $PM_{2.5}$.

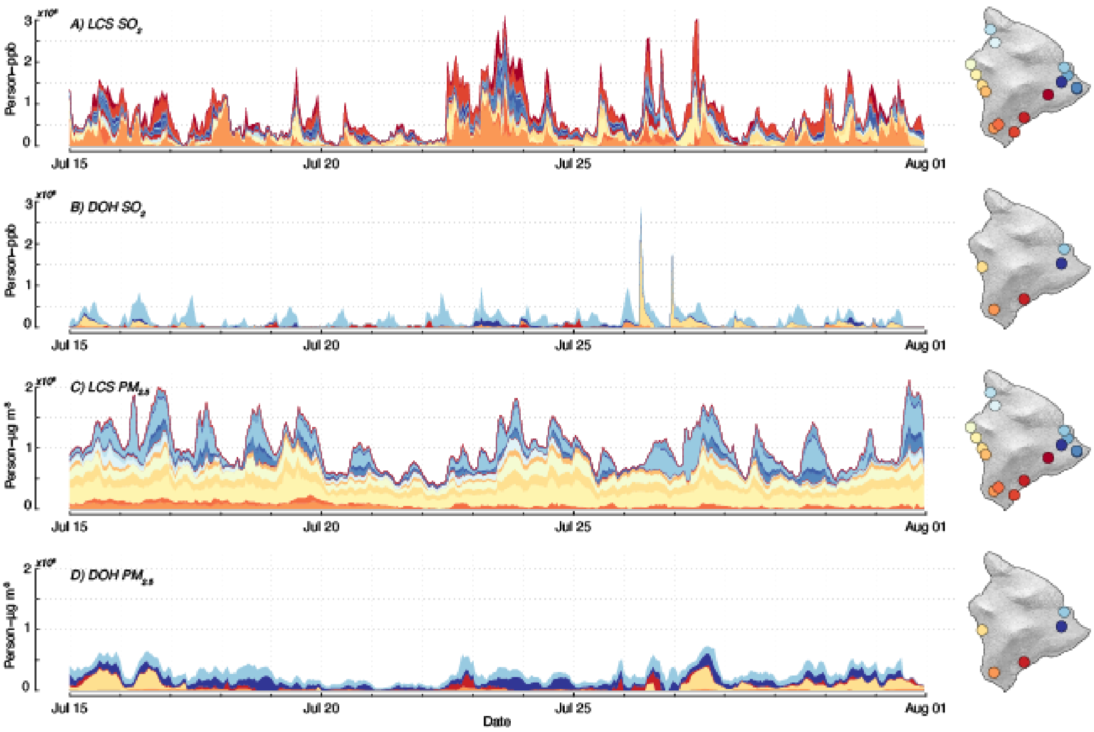

Fig S9. Hourly timeseries of cumulative population-weighted exposure (persons x concentration) for A-B) SO_2 and C-D) $PM_{2.5}$ for 16 stations from the LCS network (A,C) and five stations from the DOH network (B,D). Each unique color represents a measurement location (see map insets) and color bands are stacked so that the y-axis is the cumulative total across each respective network. The difference between networks is due to different numbers of people within a 5 km radius of network stations. Population-weighted averages, which account for this difference, are shown in Fig. S8.

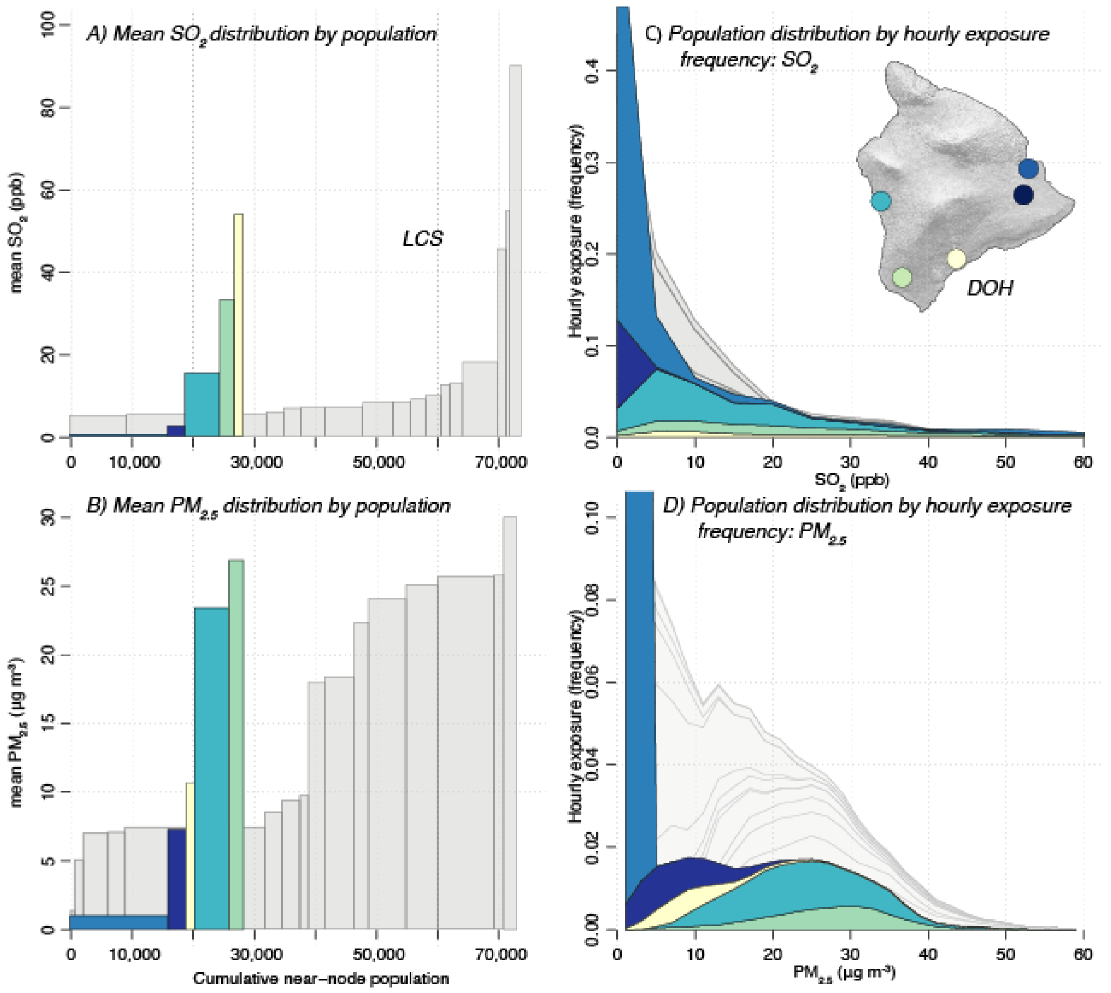

Fig S10. Population exposure to volcanic pollutants, measured by the regulatory network (DOH) over the 15-day study period (equivalent to Figure 3, but for the regulatory network rather than the LCS network). For reference, the LCS network distribution from Figure 3 of the main text is also shown as gray bars. Panels (A-B): Mean pollutant distribution as a function of cumulative near-node population (residents living within 5 km of each node). Bar width is proportional to nearby population and bar height is the average pollutant concentration measured by each node. Sensor nodes are differentiated by color, as shown on the inset map. Stations are arranged from lowest to highest average concentration. Panels (C-D): Population distribution as a function of hourly exposure frequency to SO_2 and $PM_{2.5}$. Here, the distribution of hourly concentrations experienced by each sensor node is weighted by population within 5 km of the node and arranged by average concentration.

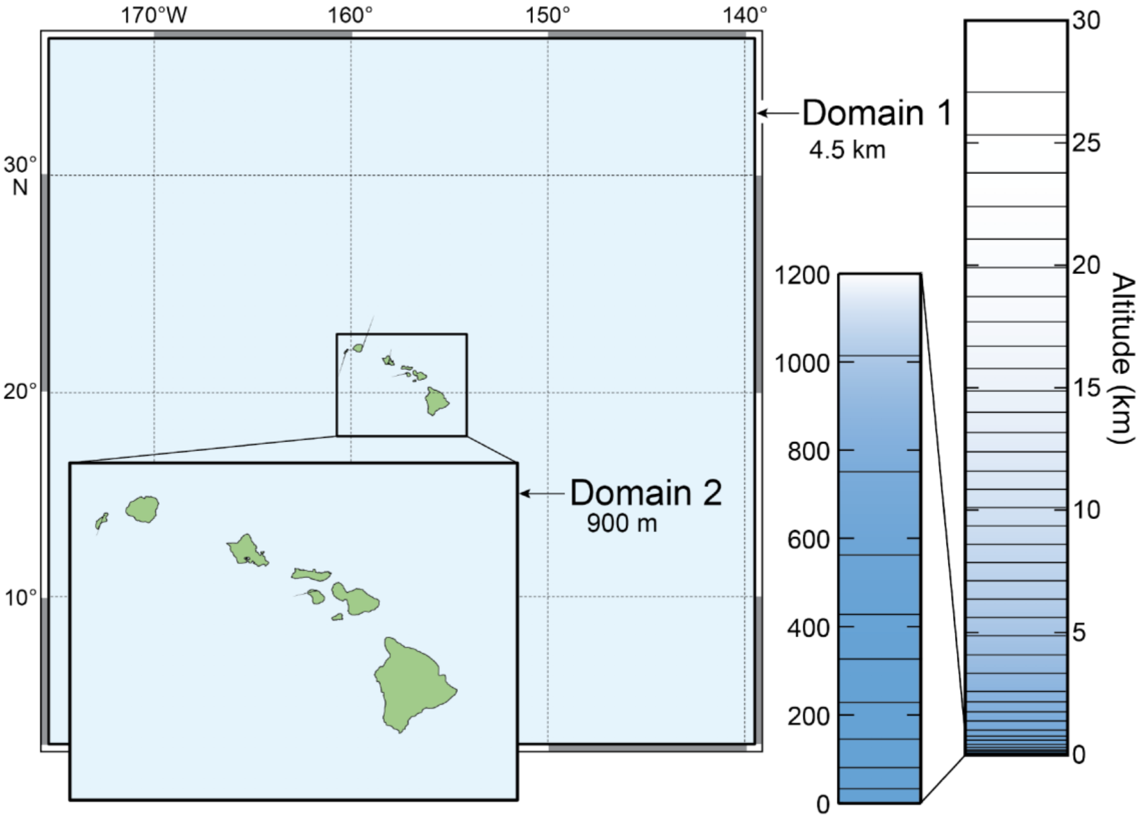

Figure S11. WRF-ARW model domains that provide meteorological input to the vog particle dispersion model. The outer domain (Domain 1) has 4.5 km resolution. The inner domain (Domain 2) has 900 m resolution and includes 51 vertical levels with a fixed top near 40 hPa.

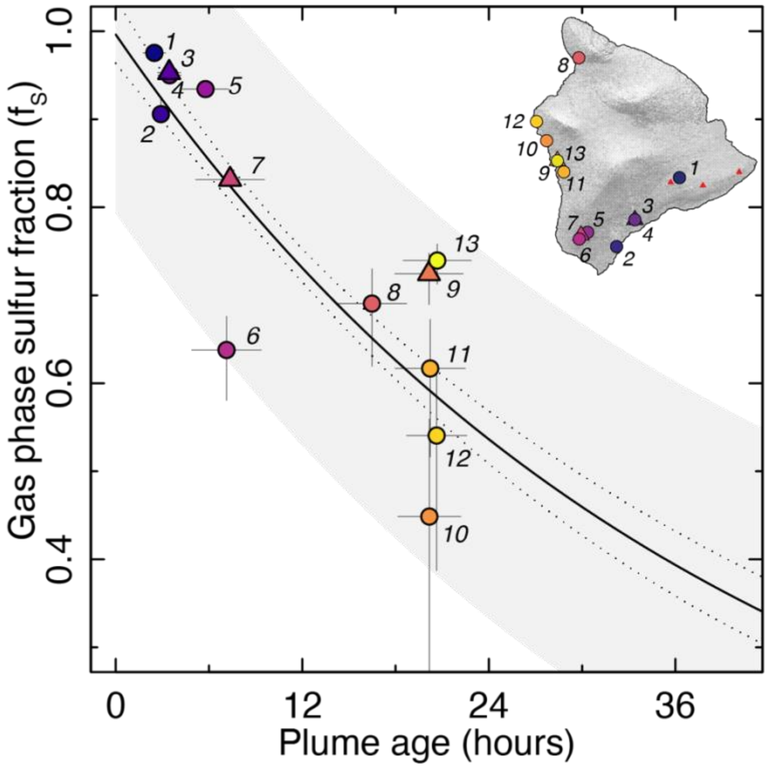

Fig. S12. Chemical evolution of the vog plume (f_S), as measured by LCS nodes (circles) and regulatory stations (triangles) downwind of the volcano. This quantifies the rate of the chemical transformation of SO_2 (gas) to sulfuric acid (PM), occurring on an estimated timescale of 7.6×10^{-6} s^{-1}. Measurement uncertainties (vertical error bars) were determined separately for SO_2 and $PM_{2.5}$ sensors during instrument calibration against reference instruments (Table S2). Plume age uncertainties (horizontal error bars) are the interquartile range of calculated parcel travel times between the LERZ and measurement location during each hour of the study period. To incorporate these uncertainties into confidence (dashed lines) and prediction (gray shading) intervals (95%), the model is fit to an array of random points uniformly sampled with the uncertainty bounds at each measurement point (n=10 points at each location, total n=130).

Table S1. All LCS network locations during the 2018 eruption. Several sensors experienced technical and/or communications difficulties and their data is not continuous during the study period. Refer to Table S2 for the sub-set of these locations with continuous data used for analysis.

	**Location**	**Longitude (°)**	**Latitude (°)**	**Elevation (m asl)**
1	Bay Clinic Health Centers, Kea‘au	-155.0396	19.62051	110
2	Big Island Processing, Kalapana	-154.9645	19.37273	38
3	Hāmākua-Kohala Health Centers, Honoka‘a	-155.4706	20.07616	372
4	Hāmākua-Kohala Health Centers, Kapau‘u	-155.7974	20.23069	136
5	Hawai‘i Academy of Arts and Science, Pahoa	-154.9445	19.50120	178
6	Hōlualoa Elementary School	-155.9482	19.61745	420
7	Hōnaunau Elementary School	-155.8792	19.45096	361
8	Innovations Public Charter School, Kailua-Kona	-155.9736	19.63188	138
9	Ka‘ū High School, Pahala	-155.4802	19.20386	297
10	Kahakai Elementary School, Kailua-Kona	-155.9778	19.61417	25
11	Kailapa Community Center	-155.8405	20.05599	36
12	Kamali‘i Road, Pahoa	-154.9007	19.44122	102
13	Kealakehe High School, Kailua-Kona	-155.9989	19.66843	93
14	Konawaena High School	-155.9159	19.50936	495
15	Laupāhoehoe High School	-155.2288	19.97967	131
16	Leilani Estates #1	-154.9094	19.45909	230
17	Leilani Estates #2	-154.9054	19.47296	197
18	Mālamalama Waldorf School, Kea‘au	-154.9690	19.55752	103
19	Miloli‘i School	-155.9073	19.18235	5
20	Mountain View Elementary School	-155.1021	19.55532	424
21	Nā‘ālehu Elementary School	-155.5792	19.06072	196
22	Nanawale Estates, Pahoa	-154.9085	19.49655	165
23	North Hawai‘i Community Hospital	-155.6637	20.02207	825
24	Ocean View Community Center	-155.7874	19.09787	658
25	Pohakuloa Training Area	-155.5356	19.75541	1,944
26	Pu‘uhonua, Pahoa	-154.9406	19.49133	205
27	Upper Ocean View	-155.7415	19.13417	1277
28	Volcano School of Arts and Sciences, Volcano	-155.2354	19.42995	1139
29	Volcanoes National Park, Kahuku Unit	-155.6779	19.06615	657
30	Volcanoes National Park, Kipukapuaulu Unit	-155.3075	19.43542	1195
31	Waiākea Elementary, Hilo	-155.0750	19.69696	38
32	Waikoloa Village	-155.7900	19.93000	289
33	West Hawai‘i Explorations Academy, Kailua-Kona	-156.0361	19.71486	29

Table S2. Measurement uncertainties (mean absolute error, MAE) for the subset of LCS network sensors shown in Figure 2, based on field co-location with regulatory reference instruments during the eruption. SO_2 sensors at several locations (*o*, *s*, *t*, *u*) failed and their statistics are not included in this table. [1]Pahala calibration period, May 27-28, 2019 with ambient SO_2 up to 800 ppb and $PM_{2.5}$ up to 30 $\mu g\ m^{-3}$. [2]Konawaena calibration period, July 7-9, 2019 with ambient SO_2 up to 50 ppb and $PM_{2.5}$ up to 35 $\mu g\ m^{-3}$. *SO_2-only node. †PM-only node using nephelometer sensor model.

Map label	Site	SO_2 (MAE, ppb)	$PM_{2.5}$ (MAE, $\mu g\ m^{-3}$)
a.	Waiakea[1]	13.6	10.3
b.	Kea`au[1]	10.7	11.1
c.	HAAS[1]	10.6	12.6
d.	Pu`uhonua[1]	10.6	2.2
e.	Mountain View[1]	12.0	3.7
f.	Volcano[2]*	7.0	4.2
g.	NPS[1]*	8.7	-
h.	Pahala[2]	3.5	3.0
i.	Na`alehu[1]	11.0	5.7
j.	Upper Ocean View[1]*†	10.3	4.8
k.	Ocean View CC[2]	2.5	3.4
l.	Honaunau[2]	3.9	3.0
m.	Konawaena[2]	7.5	3.8
n.	Kahakai[2]	3.0	3.0
o.	Kealakehe HS[2]	-	3.3
p.	WHEA[2]	3.0	2.9
q.	Waikoloa[2]	2.3	3.9
r.	Kawaihae[2]	3.7	2.9
s.	Waimea[2]	–	2.1
t.	Kapau`u[2]	-	2.9
u.	Honoka`a[2]	–	2.0
	**Average MAE**	**7.3 ppb**	**4.5 $\mu g\ m^{-3}$**

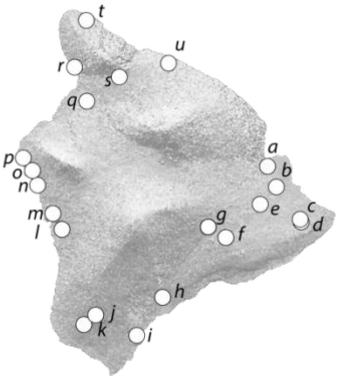

Table S3. Population (%) living within various radii of low-cost sensor (LCS, see Table S1) and Hawaii Department of Health (HDOH) networks at any point during the eruption. Spatial buffers are merged so that residents living near multiple stations are not counted twice.

Radius (km)	HDOH	LCS (Figure 3 subset)	LCS (Table S1 full set)	Combined (HDOH+LCS full set)
0.5	0.3	0.1	1.8	2.0
1	0.8	3.4	6.0	6.9
3	5.1	18.4	28.3	32.6
5	11.6	40.4	54.4	61.4
7	19.0	62.9	73.4	81.6
10	34.4	82.9	90.0	95.1

Table S4. LCS network population coverage (16 stations, see Table S2) during the July 15 – Aug 1 study period. Spatial buffers are merged so that residents living near multiple stations are not counted twice. See Figure S6 for a population distribution map.

	Upwind	Downwind	Total
People <5 km	35,486	34,928	70,414
People >5 km	58,048	45,794	103,842
Total	93,534	80,722	174,256

SI References

1. Hagan, D. H., Isaacman-VanWertz, G., Franklin, J. P., Wallace, L. M., Kocar, B. D., Heald, C. L., & Kroll, J. H. (2018). Calibration and assessment of electrochemical air quality sensors by co-location with regulatory-grade instruments. *Atmospheric Measurement Techniques* 11, 1 (January 2018): 315-328.
2. Hagan DH, Kroll JH. Assessing the accuracy of low-cost optical particle sensors using a physics-based approach. *Atmospheric Measurement Techniques*. 2020 Nov 26;13(11):6343-55.
3. Crilley, L.R., Shaw, M., Pound, R., Kramer, L.J., Price, R., Young, S., Lewis, A.C. and Pope, F.D., 2018. Evaluation of a low-cost optical particle counter (Alphasense OPC-N2) for ambient air monitoring. *Atmospheric Measurement Techniques*, pp. 709-720.
4. Petters M.D., Kreidenweis S.M.. A single parameter representation of hygroscopic growth and cloud condensation nucleus activity. *Atmospheric Chemistry and Physics*, European Geosciences Union, 2007, 7 (8), pp.1961-1971. ffhal-00296196f
5. Hafkenscheid, T., and J. Vonk. "Evaluation of equivalence of the MetOne BAM-1020 for the measurement of PM2. 5 in ambient air." (2015). National Institute for Public Health and the Environment, The Netherlands, RIVM Open Repository, https://rivm.openrepository.com/bitstream/handle/10029/558430/2014-0078.pdf?sequence=3, Accessed April 2021.
6. Businger, S., Huff, R., Pattantyus, A., Horton, K., Sutton, A. J., Elias, T., & Cherubini, T. (2015). Observing and forecasting vog dispersion from Kīlauea volcano, Hawaiʻi. *Bulletin of the American Meteorological Society*, 96(10), 1667-1686.
7. Smith, R. B., and V. Grubisic, 1993: Aerial Observations of Hawaii's Wake. *J. Atmos. Sci.*, 50, 3728-3750.
